# Handedness genetics: considering the phenotype

**DOI:** 10.3389/fpsyg.2014.01300

**Published:** 2014-11-11

**Authors:** Sebastian Ocklenburg, Christian Beste, Larissa Arning

**Affiliations:** ^1^Biopsychology, Institute of Cognitive Neuroscience, Ruhr-University BochumBochum, Germany; ^2^Cognitive Neurophysiology, Department of Child and Adolescent Psychiatry, Faculty of Medicine, Dresden University of TechnologyDresden, Germany; ^3^Department of Human Genetics, Ruhr-University BochumBochum, Germany

**Keywords:** handedness, genotype, phenotype, laterality, hemispheric asymmetry, hemispheric lateralization, motor control, ontogenesis

The question which genetic, epigenetic and environmental factors contribute to human handedness certainly is one of the central questions in research on manual asymmetries. A number of environmental factors such as season of birth (Stoyanov et al., 2011), cultural influences (Fagard and Dahmen, 2004), differential visual experience of the hands (Ocklenburg and Güntürkün, 2009; Ocklenburg et al., 2010), parental influence (Laland, 2008) and others (Schaafsma et al., 2009) have been shown to influence handedness. Moreover, several genes such as *LRRTM1* (Francks et al., 2007), *PCSK6* (Scerri et al., 2011; Arning et al., 2013), and *AR* (Medland et al., 2005; Hampson and Sankar, 2012) have been related to handedness. However, the variance in individual handedness explained by any single one of these factors is typically low, and it is not uncommon that findings in one sample cannot be replicated in others (for example see: Bloss et al., 2010; Hubacek et al., 2013). Furthermore, hardly anything is known about epigenetic and epistatic interactions between different genetic and environmental factors influencing handedness. In view of this, several authors recently argued that only a complex multifactorial model could explain the ontogenesis of handedness (e.g., McManus et al., 2013; Ocklenburg et al., 2013; Armour et al., 2014). McManus et al. (2013) estimated the number of genetic loci involved in handedness to be at least 30–40, and possibly much larger. This estimation suggests that genome-wide association studies with very large sample sizes might constitute a meaningful methodological tool to further advance our knowledge about how handedness develops. In our opinion, however, the large number of involved ontogenetic factors and likely complex interactions between them is only part of the problem why the search for the biological determinants involved in the development of handedness despite continuous research still is at a very early stage.

While a definition of handedness seems trivial at first glance, the term “handedness” actually has been used to describe a number of surprisingly different concepts, rendering clarification necessary. First, as discussed in a recent review article by Scharoun and Bryden (2014), there is an important distinction between *hand preference* and *hand performance*. *Hand preference* commonly is assessed with questionnaires such as the widely used Edinburgh Handedness Inventory (EHI, Oldfield, 1971). The EHI identifies an individual's subjectively preferred hand for 10 different manual activities (e.g., writing or striking a match). A lateralization quotient (LQ) is calculated using the formula LQ = [(R − L)/(R + L)]^*^100, with R indicating the number of activities for which the right hand is preferentially used, and L indicating the number of activities for which the left hand is preferentially used. The LQ ranges between −100 and +100, with negative values indicating a larger number of left-hand preferences, and positive values indicating a larger number of right-hand preferences. While some authors developed behavioral approaches to assess hand preference (e.g., Calvert and Bishop, 1998), the overwhelming majority of researchers uses questionnaires such as the EHI to assess hand preference.

*Hand performance*, on the other hand, typically is assessed with motor tasks such as the widely used peg board task (e.g., Annett, 1985, 2002; Scerri et al., 2011). In this task, the time participants need to move a row of 10 pegs from one side of a board to the other is measured. A quantitative value of asymmetry in hand performance is obtained by comparing reaction times for left and right hand. Other hand performance tasks include placing dots in circles or squares on a sheet of paper as quickly as possible (McManus, 1985; Tapley and Bryden, 1985), or picking up 20 matches placed on a table as quickly as possible (McManus, 1985). Interestingly, tests of hand preference and hand performance yield significantly different distributions (Peters and Durding, 1978; Nicholls et al., 2010). Hand preference typically has a J-shaped (and hence bimodal) distribution with a large number of strongly right-handed individuals, a smaller number of strongly left-handed individuals, and few individuals in between, e.g., ambidextrous to some degree, and some authors have argued that handedness in fact is a dichotomous variable (e.g., McManus, 2002; also see Corballis et al., 2012 for an overview).

In contrast, hand performance measured with the peg board task typically shows a more unimodal distribution with a shift to the right side (Annett, 1985). However, McManus (1985) has argued that the peg board data are also bimodal, and that the assumed unimodality might be an artifact of measurement noise, since a high amount of noise in the data could make it possible that a smaller distribution of left-handers is hidden in the tail of the larger distribution of the right-handers. While the details of this discussion go beyond the scope of this Opinion article, it is also important to mention that the distribution of hand performance data seems to be task-dependent to a large extent, with some tasks (e.g., McManus, 1985; Tapley and Bryden, 1985) clearly showing more bimodal distributions than the peg board task.

Although hand performance and hand preference seem to be related, the correlation between them strongly depends on the tasks used to assess the two parameters. For example, while Badzakova-Trajkov et al. (2011) found a strong correlation between hand preference and hand performance scores (*r* = 0.72), a recent study by Geuze et al. (2012), reported much lower correlation coefficients. In this study, the correlation between hand preference and peg board performance was 0.09, while it was 0.03 for hand preference and grip force, and 0.19 for hand preference and ball throwing accuracy. Moreover, even though the correlations with peg board task performance and ball throwing accuracy reached significance, none of the correlation coefficients indicated a particularly strong association. While the striking difference between the two studies may be partly due to differential percentages of left- and right-handers in the two samples (in the Badzakova-Trajkov et al., 2011, sample there were 23 left-handed, 48 mixed-handed and 64 right-handed participants while in the Geuze et al., 2012, sample there were 15 left-handed, 8 mixed-handed and 598 right-handed participants) there is clearly more research on the complex relation of hand preference and hand performance needed. Interestingly, it has also been shown that hand preference correlates with certain cognitive variables, like magical ideation and creative achievement, while hand performance does not (Badzakova-Trajkov et al., 2011). Taken together, these findings suggest that what is considered as handedness by different studies is not a uniform trait but might represent several different, distinct phenotypes.

This idea is also supported by the distinction of handedness direction and handedness consistency. Handedness direction is usually defined as the side of the preferred hand for fine motor activities, e.g., left-handed or right-handed, although some authors also use “mixed-handed” as a third category. This practice, however, has been strongly criticized by McManus (1996) who argued that mixed-handedness does not represent a natural category but rather a mixture of weak left-handers and weak right-handers. Instead, McManus (1996) suggests using a subdivision into four handedness groups (weak right, strong right, weak left, strong left) when further differentiation of handedness direction is desired. In contrast to direction, handedness consistency (some authors also use the term “handedness degree,” e.g., by Prichard et al., 2013) is the specificity of the preference for using one hand over the other, e.g., if one hand is used for all task as opposed to one hand being used for some tasks and the other hand for others. Both handedness direction and handedness consistency can be calculated based on results of a handedness preference questionnaire or a handedness performance task.

Interestingly, Arning et al. (2013) demonstrated that a sequence variation (rs10523972) in *PCSK6* was significantly associated with handedness consistency but not with handedness direction. Individuals heterozygous for a long and a short allele of an intronic 33 bp variable-number tandem repeat polymorphism were more prone to inconsistent hand preference (e.g., performing most—but not all—tasks with one hand) than individuals homozygous for a long allele. In contrast, no association between this polymorphism and handedness direction was observed. It is therefore likely that handedness direction and consistency (or strength) represent distinct phenotypes. This idea is also supported by several studies showing that handedness consistency, but not handedness direction, is a systematic predictor of performance in several cognitive domains, e.g. episodic memory retrieval, cognitive flexibility and risk perception (see Prichard et al., 2013 for a comprehensive review article).

Interestingly, the view that direction and strength of hemispheric asymmetries represent two distinct, largely independent phenotypes is also supported by recent studies in zebrafish. In this species, behavioral lateralization is modulated by structural asymmetries in the epithalamus (Barth et al., 2005; Bianco and Wilson, 2009). Genetically, the occurrence of these epithalamic asymmetries is regulated by several genes within the *NODAL* pathway which generally is relevant for the determination of left-right asymmetry in embryonic development. When expression of this pathway is symmetrical or absent, structural asymmetry *per se* is still established but its direction is not leftward like in most wildtype fish, but completely random (Concha et al., 2000). Thus, strength and direction of these hemispheric asymmetries in zebrafish likely are controlled for by two different genetic pathways. This finding is particularly interesting since Brandler and Paracchini (2014) recently suggested the *NODAL* pathway to also be involved in the ontogenesis of human handedness.

Taken together, in our opinion the large number of possibly interacting genes and non-genetic factors is only one reason why it is so difficult to determine the ontogenetic bases of handedness and other forms of hemispheric asymmetries. Another reason is that we simply do not know enough about what exactly constitutes a handedness phenotype, and how many there are. For the time being, we would like to suggest that future studies on the genetics of hemispheric asymmetries should include both a preference measure (e.g., EHI) and a performance measure (e.g., the peg board task), and that both direction and strength should be reported for those two measures in addition to a composite score such as a laterality quotient. Furthermore, research on the genetics of handedness may benefit from a stronger integration of brain activation measures, e.g., motor cortex activation differences between left- and right-handers during finger tapping or similar tasks.

## Conflict of interest statement

The authors declare that the research was conducted in the absence of any commercial or financial relationships that could be construed as a potential conflict of interest.
